# Long-Term Effect of Ciprofloxacin on Testicular Tissue: Evidence for Biochemical and Histochemical Changes

**Published:** 2013-03-03

**Authors:** Fatemeh Zobeiri, Rajab-Ali Sadrkhanlou, Siamak Salami, Karim Mardani

**Affiliations:** 1 Department of Basic Sciences, Histology and Embryology Section, Faculty of Veterinary Medicine, Urmia University, Urmia, Iran; 2 Department of Clinical Biochemistry, Faculty of Medicine, Shahid Beheshti University of Medical Sciences, Tehran, Iran; 3 Division of Molecular Epidemiology, Department of Food Hygiene and Quality Control, Faculty of Veterinary Medicine, Urmia University, Urmia, Iran

**Keywords:** Alkaline phosphatase, Ciprofloxacin, Lipid Accumulation, Lipase, Testosterone

## Abstract

**Background:**

This research studied the effect of ciprofloxacin (CPFX) on spermatogenesis. We aimed to estimate the effect of CPFX on serum levels of testosterone, LH and FSH.

**Materials and Methods:**

In this experimental study, a total of 24 mice were assigned to controlsham and test groups. We subdivided the test group into low (206 mg/kg) and high (412 mg/kg) dose CPFX groups. Control-sham animals received carboxymethyl cellulose (CMC). All animals were treated orally for 45 days. Cytoplasmic carbohydrate, lipid accumulation, cytoplasmic lipase and alkaline phosphatase (ALP) ratios were examined. Serum levels of luteinizing hormone (LH), follicle stimulating hormone (FSH ) and testosterone were measured in the control and test groups

**Results:**

The spermatogenesis cell series exhibited low numbers of cells with periodic acid Schiff (PAS)-positive cytoplasm and higher numbers of cells with lipid-positive foci. The tissue to ALP ratio and germinal epithelium (GE) lipase synthesis increased in CPFX-treated animals. In contrast to the CPFX groups, control animals showed normal cytoplasmic carbohydrate, lipid, lipase and ALP ratios in all cellular layers. In the CPFX-treated groups there was a significantly lower serum testosterone level compared with the control group. The serum levels of FSH and LH in high dosetreated animals decreased.

**Conclusion:**

Our results suggest that following long time CPFX administration major alterations occur in GE intracytoplasmic biochemistry, which may lead to loss of physiological function and ultimately result in fertility problems. CPFX is able to imbalance serum levels of gonadotropins and testosterone levels by affecting Leydig cells.

## Introduction

Due to the enhanced antibiotic resistance observed in various farm animal species, administration of antibiotics to control and/or manage microbial diseases may impose certain hazards (1). According to previous findings, a number of antimicrobial agents have been associated with damaged spermatogenesis (2). The fluoroquinolones are known as the most important group of antibiotics against different bacterial diseases in humans, poultry and animals (1, 3). Fluoroquinolones exert good bactericidal activity against a number of bacterial agents, including E. coli, Hibiscus, Pseudomonas, Staphylococcus and Chlamydia species (4).

Ciprofloxacin (CPFX) is a second-generation fluoroquinolone broad-spectrum antibiotic used to treat a number of gram-positive and -negative bacteria that cause infections of the bones and joints, and respiratory and urinary tracts. It mainly acts through inhibition of a type II topoisomerase, DNA gyrase, which is necessary to unwind replicated prokaryotic DNA. CPFX is routinely administered by urologists and fertility specialists in order to control male reproductive infections. Its side effects occur most frequently in the gastrointestinal tract and central nervous system. Allergic and cardiovascular reactions are additional adverse effects observed during treatment with CPFX (5, 6). It has been reported that CPFX significantly impairs both testicular function and structure in rats (7, 8). Following administration of CPFX, high levels of this drug were detected in prostatic tissue and seminal fluid (9). Abd-Allah et al. (10) have reported that administration of CPFX significantly reduced sperm count, motility and daily sperm production in rats, all which might adversely affect male fertility.

Leydig and Sertoli cells play key roles in spermatogenesis and cell lineage metabolism. These cells are considered to be important cells for intratesticular endocrine function (11, 12). Any disruption in their physiologic correlation with the germinal epithelium (GE) would enhance CPFX-induced damages in testicular tissue. However, the cytoplasmic biochemical alterations in GE and the role of inflammation in spermatogenesis and spermiogenesis processes are enigmatic (13, 14). Therefore the primary aim of the present study is to illustrate the histochemical alterations of cytoplasmic carbohydrate supplement, unsaturated fatty acids (lipid foci) and cytoplasmic lipase enzyme modifications. Additionally, we have evaluated tissue alkaline phosphatase (ALP) in seminiferous tubules (STs) as a biomarker enzyme for inflammation. The final aim of the present study was to determine the serum levels of testosterone, luteinizing hormone (LH), follicle stimulating hormone (FSH ) and their association with histopathological changes in the testes.

## Materials and Methods

### Animals

In this experimental study, we used 24 mature 8-week-old male NMRI mice that weighed 28.00 ± 3 g. The animals were purchased from the Animal Resources Center of the Faculty of Veterinary Medicine, Urmia University, Iran and were acclimatized in an environmentally controlled room (22+2°C, 30- 60% relative humidity, 12/12 hours dark-light cycle). Food and water were given ad libitum. In this study all experiments conducted on the animals were in accordance with the Urmia University guidance of the Ethical Committee for Research on Laboratory Animals. Following a one week acclimation period, we divided the animals into three groups (n=8), control- sham and two test groups. The test subgroups received either a high or low dose of CPFX.

### Ciprofloxacin administration

CPFX(Fluka17850, USA) was suspended in 0.5% carboxymethylcellulose (CMC) and administered by gavage once daily for 45 consecutive days. Mice in the test groups received either 206 mg/kg(low dose) or 412 mg/kg(high dose) CPFX. The 206 mg/ kg dose for mice is comparable to the human daily therapeutic dose, following correction for interspecies differences with a dose-scaling factor (15).

### Histological analyses

After 45 days the animals were euthanized by a special CO^2^ device and one-half of the testes specimens were dissected out and fixed in 10% formalin fixative for histological investigations and subsequently embedded in paraffin. Sections (5-6 µm) were stained with periodic acid Schiff (PAS) staining for intracytoplasmic carbohydrates. We evaluated the numbers of Leydig cells per mm^2^ of interstitial connective tissue and Sertoli cells per ST. The slides were analyzed under light microscope at × 400 and ×1000 magnifications.

### Histochemical analyses

In order to perform histochemical analyses, the frozen section was used for freshly dissected samples. The Sudan-Black B (SB) staining was performed to evaluate the rate of lipid foci supplement in GE for test and control-sham animals and to identify the Leydig cells cytoplasmic biosteroid supplement. ALP staining was conducted to demonstrate the ratio of this enzyme. We used lipase staining to detect the lipase enzyme ratio in GE. All specimens were evaluated at ×400 and ×1000 magnifications.

### Serum sampling and hormonal assays

Blood samples from corresponding animals were collected by decapitation. Following centrifugation at 3000 g for 5 minutes, sampleswere assessedfor serum levels of LH, FSH and testosterone. We used the enzyme-linked immunosorbent method to evaluate plasma levels of FSH, LH and testosterone.

### Statistical analysis

We analyzed the study data with SPSS software version 16 (SPSS, Inc., IL, USA). All results are presented as mean ± SD. Differences between quantitative histological and hematological data were analyzed with one-way ANOVA, followed by the Tukey test.We considered p<0.05 as significant. Correlation between lipid-positive Sertoli cells with dissociated GE in STs and the correlation between the number of Leydig cells/mm^2^ of the connective tissue with the number of Sertoli cells/ST were analyzed on an Indigo-2 O^2^ work station (Silicon Graphics, Mountain View, CA) using Matlab (Math Works, Inc., Natick, MA).

## Results

### Ciprofloxacin influenced cytoplasmic carbohydrate ratio

**Fig 1:**
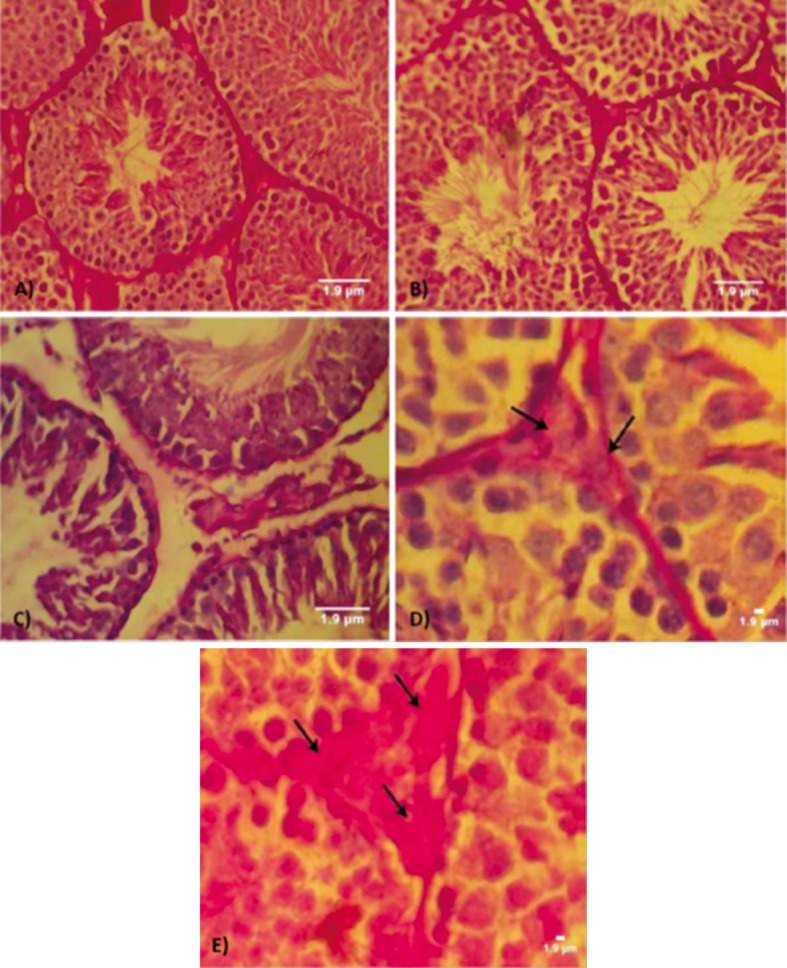
Cross-section from testes. A. Control group. Note the germinal epithelium (GE) integrity and normal PAS reaction. .B. Low dose group with light germinal cell dissociation and moderated PAS reaction present in seminiferous tubules (STs). C. High dose group with germinal cell dissociation and faint PAS-stained germinal lineage associated with remarkable edema in interstitial connective tissue. Higher magnification from interstitial connective tissue, note faint PAS-stained Leydig cells in D. and dense PAS-stained cells in E. (arrows). PAS staining, contrasted with Hematoxilineherrise, (A and B: ×400; C: ×600). Scale bars are 1.9 µm.

Light microscopic analyses showed that the spermatogonia and spermatocyte cells from low and high dose CPFX animals had low cytoplasmic carbohydrate ratios compared to control-sham animals (Fig 1A-C).

**Fig 2:**
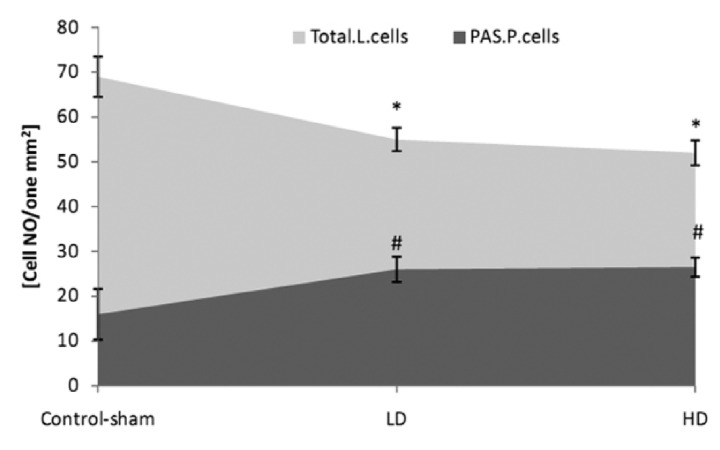
Mean number of total Leydig cells (Total.L.Cells) and PAS-positive Leydig cells (PAS.P.Cells) per mm^2^ of interstitial connective tissue. * and #;Significant differences (p<0.05) between test and control-sham groups. There are no remarkable differences(p>0.05) between low and high dose ciprofloxacin (CPFX) groups. All data are presented in mean ± SD.

In CPFX mice the majority of Leydig cells exhibited a dense PAS reaction; rarely, these cells showed faint PAS-stained cytoplasms. In CPFX-treated mice there were decreased numbers of Leydig cells/ mm^2^ of connective tissue. In contrast ,in the control animals there were significantly higher numbers of Leydig cells which were manifested with a faint cytoplasmic carbohydrate ratio (Fig 2).

**Fig 3:**
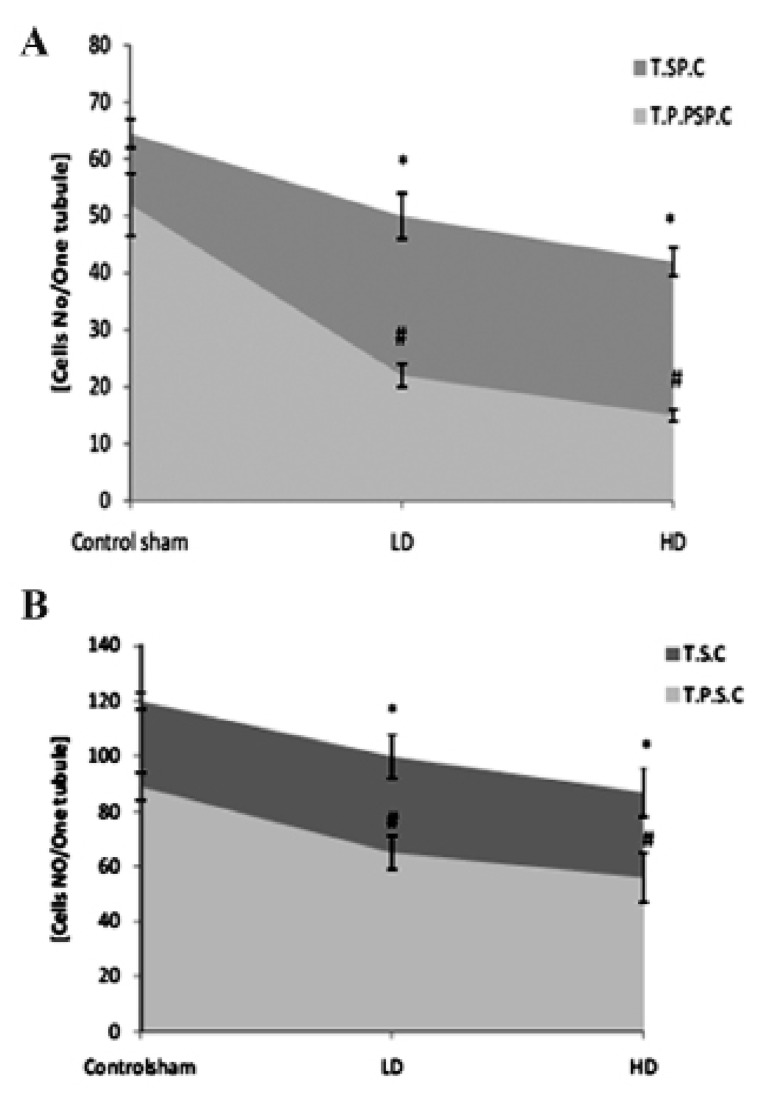
A. Average of total spermatogonia (T.SP.C) and total PAS-positive spermatogonia (T.P.P.SP.C) cells. B. Total spermatocyte (T.S.C) and total PAS-positive spermatocyte cells (T.P.S.C) per seminiferous tubule. * and #;Significant differences (p<0.05) between test and control-sham groups. There are no significant differences (p>0.05) between low and high dose ciprofloxacin (CPFX) groups. All data are presented in mean ± SD .

Comparing the number of PAS-positive cells between control-sham and test groups revealed significantly (p<0.05) lower numbers of spermatogonia and spermatocyte cells/ST which are exhibited a PAS reaction in CPFX-treated animals (Fig 3A, B).

### Ciprofloxacin altered cytoplasmic lipid accumulation

**Fig 4:**
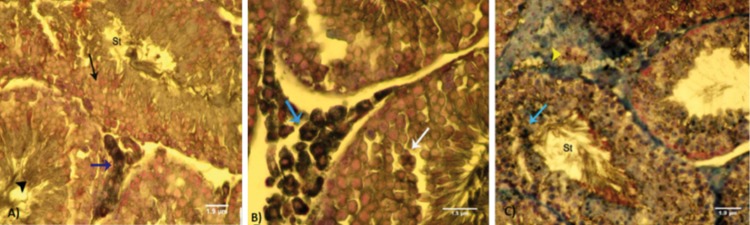
Cross-section from testes. A. Control group.Note the first three layers of the germinal epithelium (GE) with a negative reaction to lipidophilic staining (arrow); the last layers have faint Sudan black-B(SB)-stained cytoplasm (arrow head). Leydig cells (blue arrow) show dense lipid-positive cytoplasm which is indicative of normal biosteroid function. B. Low dose ciprofloxacin (CPFX)group.Leydig cells (blue arrow) show dark lipid-positive cytoplasms. Faint SB-positive stained dissociated GE shown by white arrow. C. High dose CPFX group.Note the Leydig cells that have faint SB reaction in the cytoplasm (head arrow) that is associated with remarkable edema in the interstitial connective tissue. The first three layers of germinal lineage lipid-positive reaction sites (arrow). SB staining contrasted with nuclear fast red (A: ×400, B: ×600 and C: ×400). Scale bars are 1.9µm.

Histochemical observations demonstrated that in contrast to the test groups, the first three layers of the GE in control-sham animals manifested with a faint reaction against SB staining; the upper layers had lipidophilic features. In the CPFX groups the spermatogenesis cell lineage showed remarkably higher numbers of cells with SB-positive cytoplasms (Fig 4A-C).

**Fig 5:**
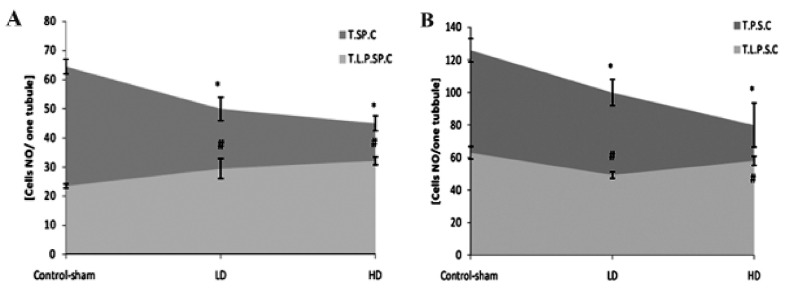
A. Average of total spermatogonia (T.SP.C) and total lipid-positive spermatogonia (T.L.P.SP.C) cells. B. Total spermatocyte (T.S.C) and total lipid-positive spermatocyte cells (T.L.P.S.C) per seminiferous tubule. * and #;Significant differences (p<0.05) between test and control-sham groups. There are no remarkable differences (p>0.05) between low and high dose ciprofloxacin (CPFX) groups. All data are presented in mean ± SD.

Test groups showed significantly (p<0.05) higher numbers of lipid-positive spermatogonia and spermatocyte cells per ST (Fig 5A, B).

**Fig 6:**
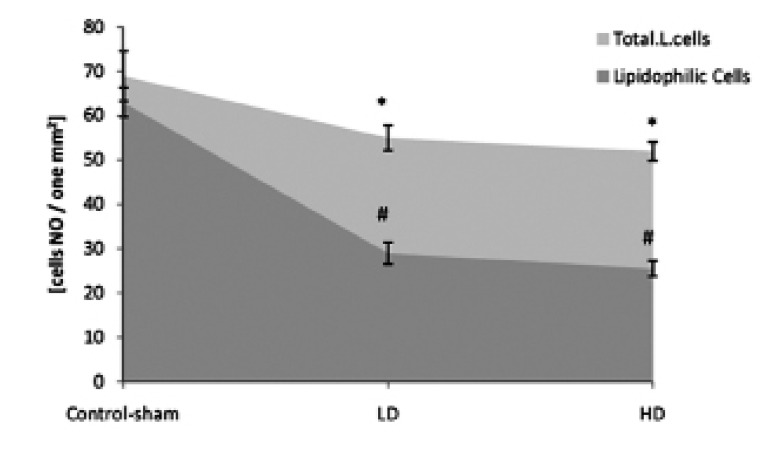
Mean numbers of total Leydig cells (Total.L.Cells) and lipid-positive Leydig cells (lipidophilic cells) per mm^2^ of interstitial connective tissue. * and #; Significant differences (p<0.05) between test and control-sham groups. There are no significant differences (p>0.05) between low and high dose ciprofloxacin (CPFX) groups. All data are presented in mean ± SD.

**Fig 7:**
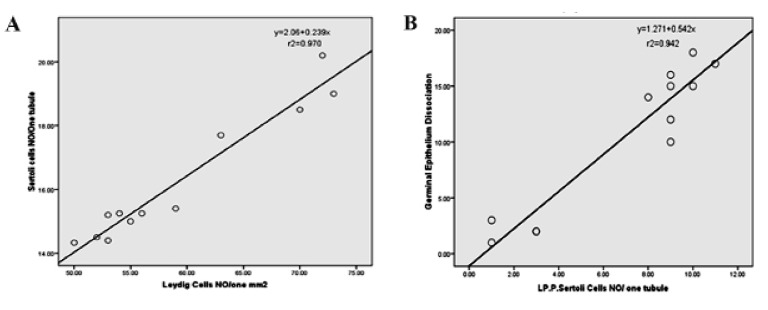
A. Correlation between number of Leydig cells in one mm^2^ of interstitial connective tissue with Sertoli cells per tubule. B. Correlation between lipid-positive Sertoli cells per tubule with germinal epithelium (GE) dissociation. There is a positive correla- .tion between the number of Leydig and Sertoli cells, Sertoli cell intra-cytoplasmic lipid accumulation and GE dissociation

In comparison to control-sham animals, CPFX-treated animals showed remarkably lower numbers of SB-positive Leydig cells/mm^2^ of interstitial tissue (Fig 6).

In both low and high dose CPFX-treated animals, higher numbers of Sertoli cells/ST showed lipid-positive reactions. Sertoli cells in controlsham animals showed a negative reaction against lipid staining. Correlation between the number of Leydig cells/mm^2^ of connective tissue with the number of Sertoli cells/ST and correlation between lipid-positive Sertoli cells with dissociated GE in STsare presented in figures 7A and B.

### Cytoplasmic lipase modification

**Fig 8:**
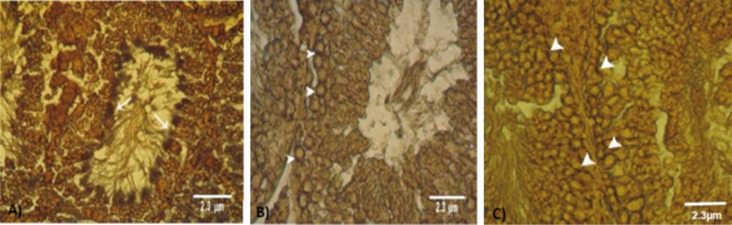
Cross-section from the testes. A. Control group. Note the faint lipase reaction in the spermiogenesis cell lineage. B. Low dose ciprofloxacin (CPFX) group and C. High dose CPFX group. Note lipase-positive areas in spermatogonia and spermatocyte cells. Lipase staining (A, B and C: ×400). Scale bars are 2.3µm.

We observed cytoplasmic lipase in spermatogenesis cells series in the control group. Animals in the test groups had high lipase-stained sites in the cytoplasms of the spermatogenesis cells series (Fig 8A-C).

### Ciprofloxacin administration elevated testicular alkaline phosphatase (ALP)

Light microscopic analyses showed significantly increased ALP-positive cells/STin CPFX animals. This impairment was mainly observed in disrupted GE.

**Fig 9:**
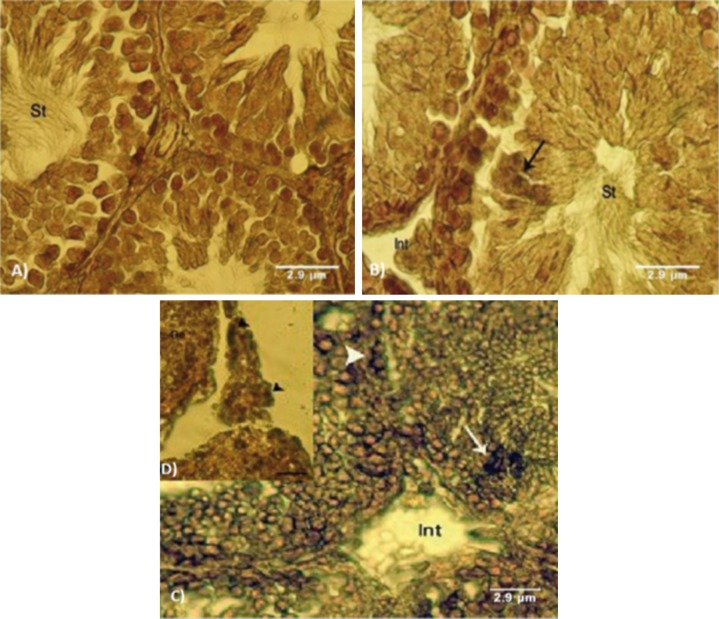
Cross-section from testes. A. Control group.Note normal seminiferous tubules (STs) with negative ALP reactivated sites in germinal epithelium (GE) and normal interstitial connective tissue (Int). B. Low dose ciprofloxacin (CPFX) group with faint edema in the interstitial connective tissue (Int) and faint ALP-stained upper layers of the GE (arrows) in seminiferous tubules (STs). C. High dosed CPFX group, not dense ALP sites in preleptotene spermatogonia cells (arrow head) and spermatocyte type one cells (arrows) associated with remarkable edema in the interstitial connective tissue (Int). ALP staining contrasted with nuclear fast red (A and B: ×600, C: ×100 and D: ×500). Scale bars are 2.9µm.

Control-sham animals showed remarkably faint reactions against ALP staining in different cell types (Fig 9A-C).

**Fig 10:**
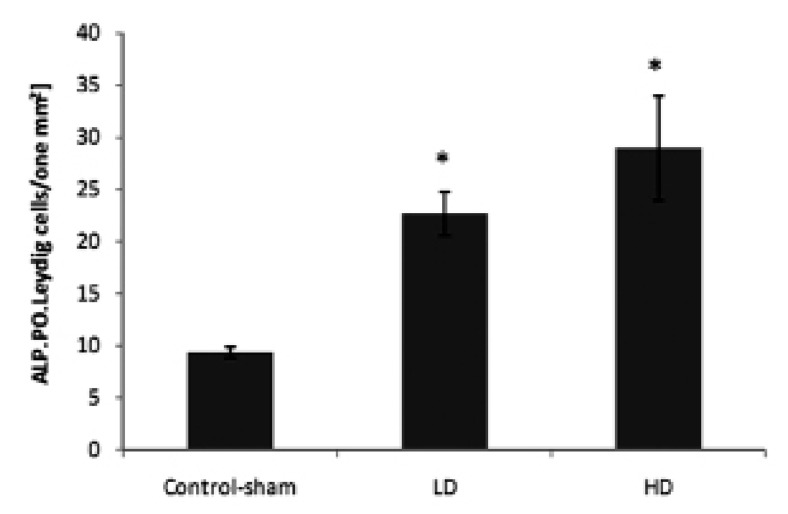
Mean average of ALP-positive Leydig cells per mm^2^ of interstitial connective tissue.Stars indicate significant (p<0.05) differences between marked groups with controlsham group. All data are presented as mean ± SD.

Significantly (p<0.05) higher numbers of Leydig cells/mm^2^ of the test animals testicleshad ALPpositive areas (Fig 10).

**Fig 11:**
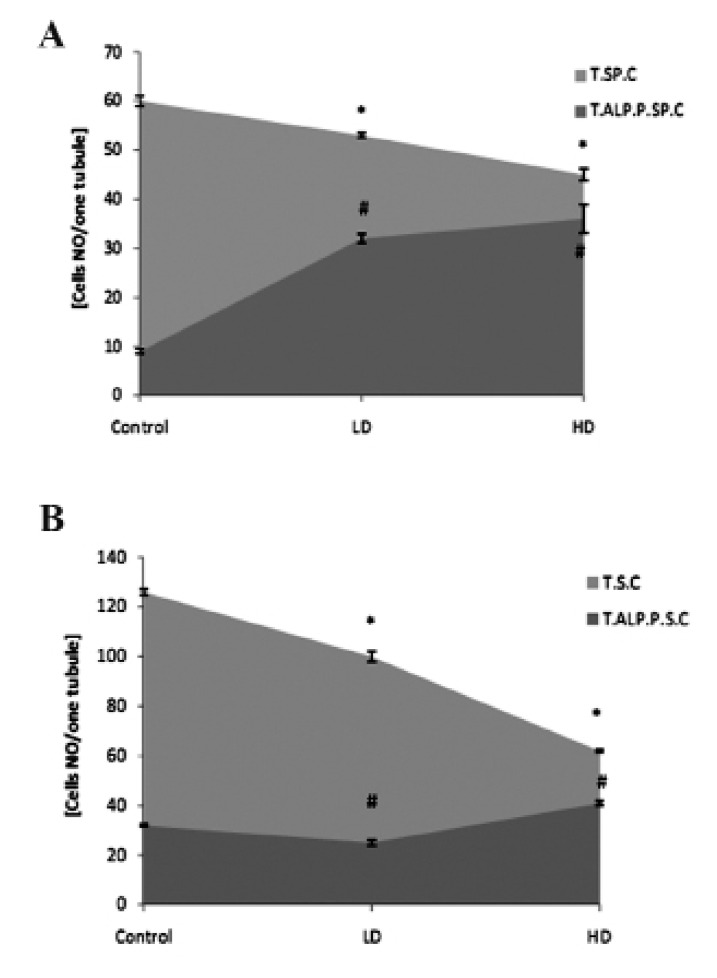
A. Average of total spermatogonia (T.SP.C) and total ALP-positive spermatogonia (T.P.P.SP.C) cells. B. Total spermatocyte (T.S.C) and total ALP-positive spermatocyte cells (T.P.S.C) per one seminiferous tubule. * and #; Significant differences (p<0.05) between test and control-sham groups. There are no significant differences (p>0.05) between low and high dose ciprofloxacin (CPFX) groups. All data are presented in mean ±SD.

Test groups showed significantly (p<0.05) higher numbers of ALP-positive spermatogonia and spermatocyte cells per ST (Fig 11A, B).

### Ciprofloxacin affected serum levels of testosterone, LH and FSH

Hematological analyses revealed that the testosterone level decreased in CPFX-treated mice, which was statistically significant (p<0.05) between different groups. The serum levels of FSH and LH in high dose-treated animals decreased (p<0.05; Table 1).

**Table 1:** Effects of ciprofloxacin (CPFX) on serum levels of testosterone, FSH and LH


Groups	Testosterone(ng/ml)	FSH (Iu/L)	LH (Iu/L)

**Control**	5.42 ± 0.23^a^	0.69 ± 0.09^a^	0.76 ± 0.11^a^
**Low dose**	4.58 ± 0.18^b^	0.77 ± 0.15^a^	0.76 ± 0.10^a^
**High dose**	4.2 ± 0.10^c^	0.53 ± 0.09^b^	0.60 ± 0.11^b^


Data are presented as mean ±SD. Different letters in each column indicate that data are significantly different (p<0.05).

## Discussion

Although the therapeutic and prophylactic effects of CPFX on different gram-positive and -negative bacteria has been well documented, various studies reported that even short term administration of CPFX promoted male reproductive toxicity (8, 10). Several reports indicated that the therapeutic dose of CPFX in prolonged and short time consumption remarkably decreased sperm hyper activation and motility (16, 17). Although it has been reported that CPFX in therapeutic dose resulted in considerable apoptosis of spermatogonia cells (17), the exact mechanism and/or pathophysiology for CPFX-induced damages in testicular tissue has not been determined.

Our findings showed that long term administration of CPFX resulted in remarkable alterations in intracellular biochemical supplements. The intracytoplasmic carbohydrate ratio decreased in spermatogenesis cell lineage and simultaneously the lipid foci supplement and lipase enzyme increased in the cytoplasm of these cells. An increase in ALP level was mainly manifested in degenerated GE. Significant changes also occurred in serum levels of testosterone, LH and FSH.

Previous studies have shown that glucose transporters are the main ways to transfer glucose to the STs; carbohydrates are the major sources of energy for hyper-mitotic cells (18). Thus any degeneration could result in interruption of glucose passage to the STs and ultimately to GE (12).

In the present study, both spermatogonia cells and spermatocytes had PAS-negative reactions in CPFX-treated animals. Thus, following CPFX administration, there was decreased glucose transport and/or metabolism in spermatocytogenesis and the spermatogenesis cell lineage which resulted in a switch in their energy source from glucose to lipids. As a result, cytoplasmic lipid foci increased in the cytoplasms of these cells, particularly in the first three layers. Simultaneously, due to insufficient energy, these cells were unable to synthesize essential proteins and therefore they underwent apoptosis and disruption (19, 20). Since there were no significant differences in studied parameters between low and high dose CPFX-treated animals, we concluded that CPFX had the capability to impact the cellular biochemical supplements, even at in low doses.

The increase of lipase enzyme synthesis in CPFX-treated groups revealed that the metabolic pathway of the cell lineage in the first three layers of the GE was altered by using the lipid sources from different biological activities. The number of Sertoli cells with SB-positive cytoplasm increased in CPFX-treated groups which showed the effects of CPFX in lipid accumulation in Sertoli cells. Lipid supplement in Sertoli cells varies with different conditions. Forinstance during phagocytosis of residual bodies or damaged cells, the intracytoplasmic ratio of lipids increases in their cytoplasm (12, 21). Our ALP staining was in good accordance with obtained results as the testes of the CPFX-treated animals exhibited strong ALP reactions, particularly in the disrupted cells. According to these findings it would be more logical to say that the number of ALP-positive disrupted GE cells increased in STs with an eventual elevation in phagocytosis.

According to previous reports, constant levels of FSH and LH are essential for initiating and supporting the spermatogenesis process (22, 23). Hence degeneration of Sertoli and Leydig cells in the CPFX-treated groups may be associated with severe alterations in serum LH and FSH levels. On the other hand, Leydig cells control Sertoli cell physiological bioactivities by the synthesis of testosterone (22, 23). FSH increases the synthesis of androgen binding protein by Sertoli cells which is needed to maintain high concentrations of testosterone (22, 24). Our histochemical findings confirmed the above reports. The increase in the number of Leydig cells with positive ALPstained cytoplasms and the decreased number of cells with lipidophilic cytoplasm in CPFX groups suggested that the biosteroid activity of the Leydig cells reduced in CPFX-dosed animals. Thus, we concluded that remarkable degeneration of Leydig cells after administration of different doses of CPFX resulted in significant reduction in serum testosterone levels; this reduction in testosterone was responsible for the Sertoli cell degeneration and consequent GE disintegration.

## Conclusion

Following CPFX administration the spermatogonia, spermatocyte and Sertoli cells in STs switch their energy source from glucose to lipids. Thus, inadequate energy supplement leads to cellular degeneration. Impairment of Leydig cells in testosterone synthesis negatively impacts this pathological process by affecting Sertoli cells.
